# *Pseudomonas* spp. diversity is negatively associated with suppression of the wheat take-all pathogen

**DOI:** 10.1038/srep29905

**Published:** 2016-08-23

**Authors:** Zia Mehrabi, Vanessa E. McMillan, Ian M. Clark, Gail Canning, Kim E. Hammond-Kosack, Gail Preston, Penny R. Hirsch, Tim H. Mauchline

**Affiliations:** 1 Institute for Resources Environment and Sustainability, University of British Columbia, Vancouver, BC V6T 1Z4 Canada; 2 Long Term Ecology Laboratory, University of Oxford, South Parks Road, Oxford, OX1 3PS, UK; 3 Department of Plant Biology and Crop Science, Rothamsted Research, Harpenden, AL5 2JQ, UK; 4 Department of AgroEcology, Rothamsted Research, Harpenden, AL5 2JQ, UK; 5 Department of Plant Sciences, University of Oxford, South Parks Road, Oxford, OX1 3RB, UK

## Abstract

Biodiversity and ecosystem functioning research typically shows positive diversity- productivity relationships. However, local increases in species richness can increase competition within trophic levels, reducing the efficacy of intertrophic level population control. *Pseudomonas* spp. are a dominant group of soil bacteria that play key roles in plant growth promotion and control of crop fungal pathogens. Here we show that *Pseudomonas* spp. richness is positively correlated with take-all disease in wheat and with yield losses of ~3 t/ha in the field. We modeled the interactions between *Pseudomonas* and the take-all pathogen in abstract experimental microcosms, and show that increased bacterial genotypic richness escalates bacterial antagonism and decreases the ability of the bacterial community to inhibit growth of the take-all pathogen. Future work is required to determine the generality of these negative biodiversity effects on different media and directly at infection zones on root surfaces. However, the increase in competition between bacteria at high genotypic richness and the potential loss of fungal biocontrol activity highlights an important mechanism to explain the negative *Pseudomonas* diversity-wheat yield relationship we observed in the field. Together our results suggest that the effect of biodiversity on ecosystem functioning can depend on both the function and trophic level of interest.

Biodiversity has both positive and negative effects on ecosystem functioning^1^,^2^. These different effects are known to occur locally from competitive dominance of species that have a high or low functional output and from complementary or antagonistic interactions that increase with species richness^3^,^4^,^5^. Positive effects of biodiversity on productivity and resource capture within trophic levels are most commonly reported in the literature, but for practical management of ecosystems it is important we understand the range of conditions under which diversity causes negative effects on functioning. This is particularly relevant in conventional agriculture, where locally antagonistic interactions, such as between weeds and crop plants, have underpinned the rationale for the creation of species-poor production systems. However, lack of research, alongside potential mismatches between ecosystem functions and ecosystem services highlight the large gap in the practical application of current biodiversity and ecosystem functioning research to agriculture^2^,^6^.

Crop diseases pose some of the greatest challenges to food security of the 21^st^ Century^7^,^8^,^9^,^10^. Root-associated soil bacteria might offer potential solutions to this problem by combating pathogens with anti-microbial compounds and improving plant growth^11^,^12^. However, a limiting factor to this solution is that rhizosphere bacteria can compete with each other with a range of antibacterial agents^13^,^14^,^15^. Whilst the spatial dynamics of antibiotic production in the rhizosphere are poorly understood, bacteria which possess the ability to control fungal pathogens can be rendered less effective at biocontrol if they are defeated by other bacteria^16^,^17^,^18^. This means that in agricultural crops where fungal pathogens are problematic, reduced crop yield may result from increased bacterial richness of potential biocontrol genotypes. An open question remains as to (1) the extent to which bacterial diversity patterns in soils conducive to fungal pathogens match this expectation, and (2) the degree that other aspects of bacterial diversity, such as evolutionary relatedness or resource use overlap, might alleviate the intensity of competition between bacteria^19^,^20^.

Take-all disease is caused by a pervasive fungal pathogen (*Gaeumannomyces graminis* var. *tritici*) in wheat (*Tritium aestivum*) and leads to devastating yield losses^11^,^12^. Populations of antifungal *Pseudomonas* spp. bacteria have been shown to naturally control take-all, but only after a period of yield losses lasting 4–6 cropping seasons^11^,^12^. However, a novel phenomenon was recently discovered in which particular wheat varieties are able to limit the build up of take-all in a single season and create disease suppressive soils prior to an epidemic taking place^21^. The putative role of some *Pseudomonas* spp. in take-all control, and their general dominance in the soil bacterial community, positions them as a useful focal group taxon to explore the phenomenon of take-all build up, and to test more general ideas of how bacterial diversity in wheat root system might be related to fungal pathogen suppression.

We used field trials to test if the genotypic richness *Pseudomonas* spp. in wheat root systems is correlated to severity of infection by the take-all pathogen and yield loss (e.g. Fig. 1). We pre-cultured field plots with different wheat varieties to generate treatments for high or low build up of the take-all inoculum^21^, and undertook our field assays in the following season on genetically identical clones of a commercial wheat variety (*T. aestivum* var. Xi19). We tested for generic changes in the background rhizosphere bacterial community structure based on relative abundance of 16S rRNA genes and then sub-sampled culturable isolates in both the endosphere and rhizosphere of wheat roots to describe the patterns of *Pseudomonas* spp. genotypic richness and phylogenetic diversity across pre-culture treatments at finer resolution.

We then set up a series of abstract *in vitro* experimental model microcosms to test for a causal effect of *Pseudomonas* genotypic richness on growth inhibition of the take-all pathogen. We orthogonally partitioned the effect of *Pseudomonas* genotypic richness with *Pseudomonas* phylogenetic diversity (based on the *gyrB* housekeeping gene) and *Pseudomonas* functional trait diversity (based on carbon use profiles) to separate the effects of each component of diversity on the sign of bacterial interactions^20^,^22^. We then conducted a complementary set of competition experiments to test if the effects of *Pseudomonas* genotypic richness on inhibition of the take-all pathogen could be explained by the degree of antagonism between bacteria expressed in *Pseudomonas* mixtures (defined here as the compound effects of direct antagonistic toxin production and indirect exploitative resource competition).

## Methods

### Field trials

The field trial was undertaken on the Rothamsted Research experimental farm UK (N51°48′27″, W0°21′44″) during 2010–2012. A commercial winter wheat variety (Xi19) was sown in the autumn on soils in 2011, that in 2010, had been pre-cultured either high (Hereward) or low (Cadenza) take-all inoculum ‘build up’ wheat varieties^21^. Seeding was at 350 seeds m^−2^ on each of eight 10 m × 3 m plots (4 replicates × 2 soil culture treatments). Take-all disease intensity on wheat was characterized with the take-all intensity index (TAI)^23^. To determine the diversity of the dominant members of *Pseudomonas* spp. we randomly sampled 5 roots systems from each plot (plants were at late milk stage), pooled the roots, and extracted bacteria from both the rhizosphere soil and the root endosphere (after washing roots and surface sterilizing with hypochlorite followed by ethanol^24^). We pooled the roots (1 pool per plot, each containing 5 root systems), to obtain plot-level estimates of the dominant members. One caveat of this sampling procedure is that the details of alpha diversity at level of the root system may have been lost in place of plot level bacterial beta-diversity. Extracts were dilution plated onto *Pseudomonas* selective agar with CFC antibiotics (Oxoid) and then incubated for 96 h at 24 °C. Fifteen single colonies (or the maximum if less) from both the endosphere and rhizosphere of each plot were randomly sub-sampled and DNA from each colony was extracted by cell lysis with MicroLysis + (Microzone, UK) (Total isolates = 174, 91 from Hereward and 87 from Cadenza plots, see Table S1). This sub-sampling and selection procedure did not attempt to represent the full diversity of the endosphere and rhizosphere, but provide a feasible representation of the diversity of most dominant culturable isolates, with the caveat that more ‘rare’ strains may have been overlooked. Bacterial genotype was determined by genetic fingerprinting of enterobacterial repetitive intergenic consensus sequences (ERIC)^25^. For unique ERIC profile genotypes, we PCR amplified, sequenced and aligned a 940 bp fragment of the DNA Gyrase subunit B (*gyrB*) gene for each distinct genotype present in the study^26^ and perfomed maximum-likelihood based estimation using RAxML (v.7.0.4), optimized under the GTR-GAMMA substitution model^27^. We define phylogenetic diversity (PD) throughout the study as the total phylogenetic branch length of the *gyrB* sequence-determined genotypes present in a given assemblage^28^. Importantly, we used this gene only as a phylogenetic proxy, and did not perform a full multi-locus sequence analysis, as would be required for more in depth phylogenetic analysis. The sequence data generated in this project have been deposited in the GenBank Nucleotide database (accession numbers *KF059880-KF059937*). The field trial was combine-harvested and fresh grain weights of Xi19 recorded per plot at grain maturity in August 2012. Grain dry matter was determined by oven-drying 80 g sub-samples of the fresh grain for 16 h at 105 °C. Grain yields were then adjusted to 85% dry matter and scaled to tonnes ha^−1^.

### Fungal pathogen inhibition

**Figure 2:**
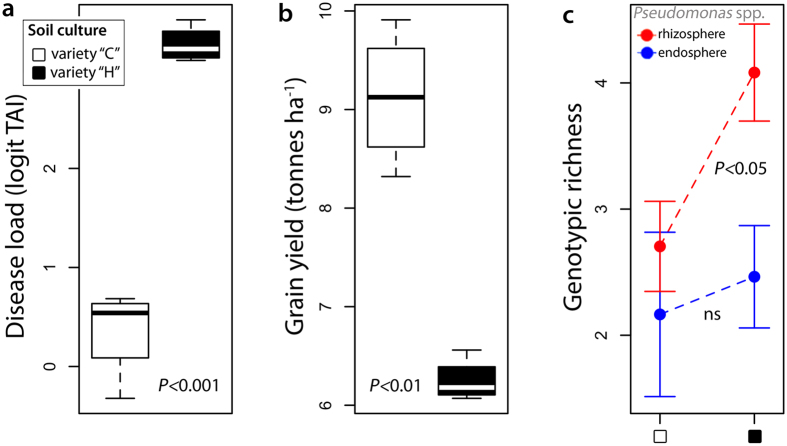
Increased bacterial richness is associated with increased fungal disease load (TAI) and wheat yield losses in the field. (**a**), wheat variety choice in the first year of cropping directly influences the intensity of take-all disease in the following isogenic wheat crop (cv. Xi19). (**b**), this has a marked effect on grain yield. (**c**), these negative effects of the take-all fungal pathogen are associated with an increased rarefied genotypic richness of *Pseudomonas* spp. in the rhizospheres of wheat plants (data show means ± s.e.m). (174 isolates in total; Cadenza plots = 87, Hereward plots = 91). All comparisons are made with Welch’s t-test.“ns” = non-significant. “C” = Cadenza; “H” = Hereward; TAI = take all intensity index (see METHODS for details). In all cases n = 4.

We conducted a biodiversity ecosystem functioning experiment to determine how increasing genotypic, phylogenetic and functional diversity of *Pseudomonas* spp. influenced inhibition of take-all fungus (*Gaeumannomyces graminis* var. *tritici*). Eight isolates (Fig. 2) representing the phylogenetic breadth of genotypes in our *Pseudomonas* spp. library were grown up in Lysogeny broth (LB) at 25 °C for 12 h, shaken at 200 rpm. Late exponential phase bacteria were then pelleted by centrifugation, washed twice in 1× phosphate buffered saline (PBS), incubated for 6 h at room temperature to allow terminating division cycles and adjusted to an OD_600_ of 1.0, with 1 × PBS. We assembled the genotypes into 49 mixtures in a substitutive design (e.g at constant density), randomly constructing 20 assemblages representing a gradient of phylogenetic diversity within each of the 2 and 4 genotype richness treatments (8 × 1 genotype; 20 × 2; 20 × 4 genotypes; 1 × 8 genotypes = Total 49). To do this first the phylogenetic diversity of all possible combinations of 2, or 4 genotypes was computed, and then 20 assemblages with unique phylogenetic diversity values were randomly sampled from each pool. This design allowed us to orthogonally test for the role of phylogenetic diversity vs. genotypic richness in inhibition of the fungus. Eight microliters of each community (at OD_600_ 1.0) was spotted 2 cm from the edge of Petri dishes containing potato dextrose agar (PDA, Oxoid^TM^, pH 5.6), and incubated at 27 °C for 48 hours. We then placed a 5 mm plug from the leading edge of a culture of the take-all pathogen (grown for 7 days at 24 °C) in the center of the dish, and plates were incubated for a further 7 days at 24 °C before photographing at a standardized height. All plates were spotted with a negative control of PBS, which had no effect on fungal growth. Photographs were analyzed using Fiji^29^, and inhibition measured as the total pixels free from fungal growth. Functional traits relevant to resource use were proxied by measuring the differential ability of each isolate to utilize 95 different carbon substrates. Assays were completed using GN2 MicroPlates (Biolog, Inc.), with 100 μL of each OD_600_ adjusted population per well, incubation at 25 °C for 72 h and measurement at OD_590_. We define functional diversity (FD) as: 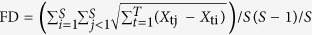 where T is the total number of traits, X_ti_ and X_tj_ are the values of the trait “t” of genotypes “i” and “j” and S is the total number of genotypes in a given assemblage^30^. The experiment and functional trait assays were repeated in triplicate.

### Bacterial antagonism

To assess how different levels of genotypic, phylogenetic or functional diversity influenced antagonistic interactions between bacteria, we conducted a second biodiversity ecosystem functioning experiment. Here we used 45 of the same assemblages used in the take-all pathogen inhibition experiment (8 × 1 genotype; 19 × 2 genotype; 19 × 4 genotypes; 1 × 8 genotypes = Total 45; with a reduction to 19 for 2–4 genotypes due to practicalities of 48 wells per replicate) and cultured (from an initial density of ∼10^7^ cells ml^−1^) them in LB broth at 27 °C for 18 h, shaken at 200 rpm (Note: LB is a rich medium which we used to match initial culture conditions and accelerate growth, future work might also repeat this with 1/10^th^ Tryptone Soya Broth or root exudates). We centrifuged each mixture and deposited 150 μl of the cell free supernatant into separate wells of a microtitre plate. We then overlaid 50 μl of OD_600_ adjusted populations of each of the 8 different genotypes, subjecting each genotype to the compound legacy effects of antagonistic toxin production and nutrient depletion by each community. Productivity differences were proxied as simply OD_600_ after 6 h incubation at 25 °C, shaken at 200 rpm. OD scales well to dry weight biomass and is a useful proxy for integrated productivity (i.e. the product of cell sizes and counts)^31^,^32^. The whole experiment was repeated in triplicate.

### Statistical analysis

To determine the differences between take-all disease intensity (logit transformed), log total grain yield and bacterial diversity (genotypic richness/phylogenetic diversity) in our field plots we used Welch’s t-test. We rarefied phylogenetic diversity measures to account for species differences between plots, using the standalone phylorare() function^33^ in R 3.0^34^. We also used individual-based rarefaction on genotypic richness in each field plot to account for sampling effort biases that resulted from plots that did not yield 15 single separate colonies^35^. Analysis of Similarity^36^ based on presence-absence data and Bray-Curtis distances were used to test for differences in community composition between plots. We used linear models, to orthogonally test for the effect of genotypic richness and phylogenetic diversity on inhibition of take-all and bacterial antagonism in the 2–4 genotypic richness assemblages. Genotypic richness was fitted as a categorical variable first in all the models and phylogenetic (or functional) diversity fitted after as a continuous variable, with first order interactions. As no significant interactions were present in any of the models, we removed the interaction terms and assumed fixed slope estimates for the models presented in the main text. The biodiversity metrics we used as our response were characterized using the net biodiversity effect^37^: log(Φ_obs_/Φ_exp_), where, for the fungal inhibition experiment, Φ_obs_ is the overall inhibition of a given assemblage, and Φ_exp_ is the mean inhibition from each genotype present in the assemblage in monoculture.; and where, for the bacterial inhibition experiment, Φ_obs_ is the overall productivity of a genotype on the spent medium from a given assemblage, and Φ_exp_ is the mean productivity of that same genotype on the spent medium from each genotype present in the assemblage. All models were run and assumptions validated in R^38^.

## Results

### Field trials

In our field trials we found that, the take-all severity on wheat roots of isogenic *T. aestivum* var. Xi19 was dependent on the wheat variety used in soil pre-culture treatments (Welch’s t-test, *t*_*3.91*_ = 11.64, *P* < 0.001; Fig. 2a). This difference in disease load was related to large differences in overall grain yields of wheat (31% ± 4% SE [~3t ha^−1^] higher yields in plots with low take-all disease) (Welch’s t-test, *t*_*4.24*_ = 9.26, *P* = 0.0006; Fig. 2b). To investigate whether this difference in disease load and yield was associated with *Pseudomonas* spp. we sampled *Pseudomonas* communities in the endosphere (inside) and rhizosphere (outside) of the roots of the isogenic wheat plants, and then compared genotypic richness and phylogenetic diversity across plots. We found that the plots with highest take-all disease loads, and lowest wheat grain yields, contained the highest genotypic richness of *Pseudomonas* in the rhizosphere (Welch’s t-test, *t*_*5.98*_ = 2.63, *P* = 0.039; Fig. 2c). We did not detect clear differences in rhizosphere *Pseudomonas* community composition *per se* between plots with high or low disease load (Analysis of Similarity, Bray-Curtis, R = 0.19, *P* = 0.08). We found no differences in phylogenetic diversity of *Pseudomonas* in the rhizosphere of wheat roots between low or high take-all plots (Welch’s t-test, *t*_*4.96*_ = 0.40, *P* = 0.70; Fig. S5). Endosphere *Pseudomonas* genotypic richness or phylogenetic diversity also showed no significant relationships with take-all intensity or yield (Fig. 2, Fig. S5). On average 1.7% of 16S rRNA reads were identified as *Pseudomonas* across the study plots. *Pseudomonas* relative abundances and generic microbial community composition based on 16S rRNA sequences did not show obvious differences between treatments (Fig. S6. Supplementary Notes 1).

### *In vitro* experimental isolates

**Figure 3:**
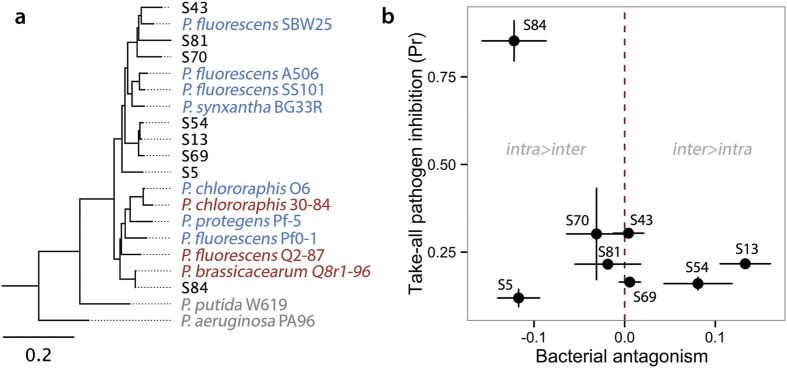
*Pseudomonas* spp. used in the experiments. (**a**), phylogenetic relationships between the genotypes used in the *in vitro* experiments and other well known biocontrol genotypes based on maximum likelihood tree estimated on a 940 kb fragment of the universal houskeeping gene *gyrB*, using the RAxML algorithm (with GTR-GAMMA substitution). Scale bar represents nucleotide substitutions per site. Blue strains are known biocontrol agents, red strains were previously isolated from wheat root systems and have been implicated in take-all control, grey strains are outgroups^38^. (**b**), Take-all pathogen inhibition and bacterial antagonistic ability of the strains. Take-all pathogen inhibition is the area of fungal mycelial growth inhibited by each *Pseudomonas* genotype in Petri dish assays (1 = no fungal growth, and 0 = complete fungal growth). Bacterial antagonism is the log ratio of strain productivity (OD _600_) on spent liquid medium cultured by its own genotype versus its mean productivity on spent medium cultured by all other genotypes. Data show means ± s.e.m. n = 3.

The relationship between the genotypes we used in our *in vitro* experiments, and other well-known biocontrol *Pseudomonas* genotypes is given in Fig. 3a. Our genotypes expressed differences in *in vitro* take-all pathogen inhibitory activity and intragenotypic vs. intergenotypic bacterial antagonism. However, we did not observe any genotype level trade-off between take-all pathogen inhibitory ability and bacterial antagonism. The most potent inhibitor of the take-all pathogen (S84: 85% ± 6% SE inhibition of mycelial growth), nevertheless expressed the classic self-limiting trait where intragenotypic antagonism > intergenotypic antagonism (Fig. 3b). Strain S84 also clustered with other antibiotic producing strains *P. brassicacearum* Q8r1-96 and *P. fluorescens* Q2-87, previously implicated in control of the take-all fungal pathogen^39^. We found no correlation between pairwise distances in carbon resource use profiles and phylogenetic distances based on *gyrB* (Mantel test, *P* = 0.74; Fig. S2).

### Fungal pathogen inhibition

**Figure 4:**
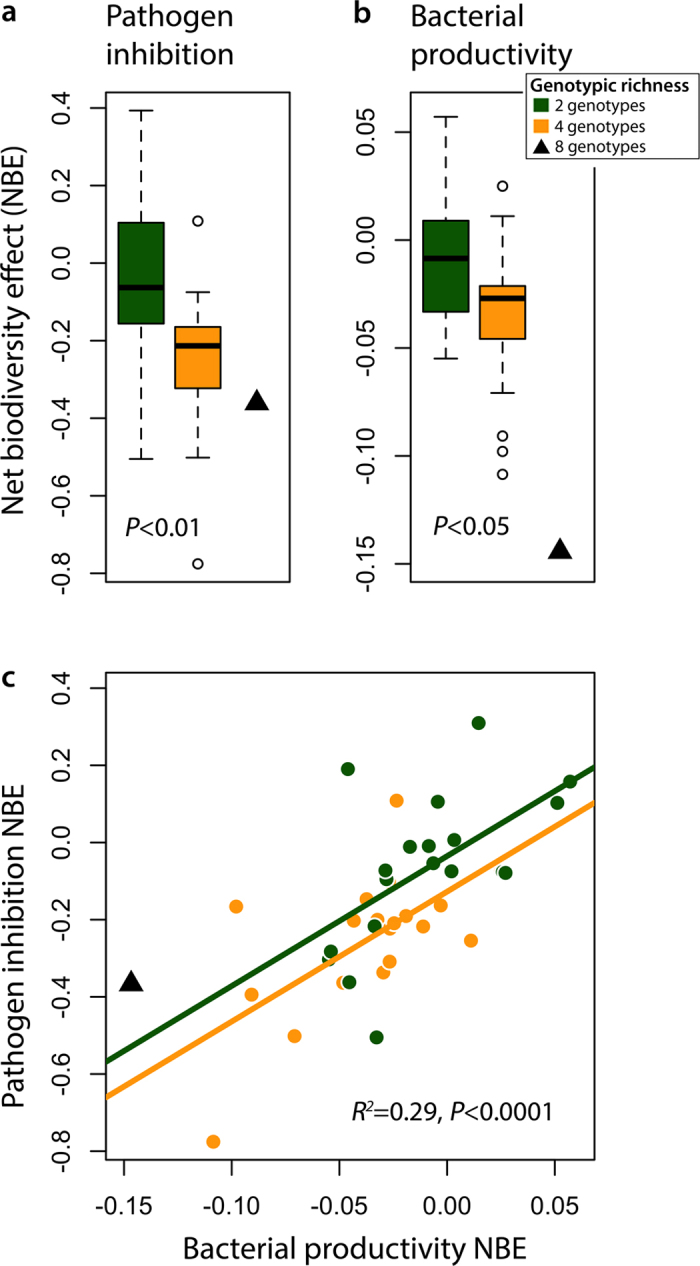
Negative effects of bacterial genotypic richness on fungal pathogen inhibition result from increasing intergenotypic antagonism between soil bacteria. (**a**), net negative effects of increasing *Pseudomonas* genotypic richness on inhibition of wheat take-all fungal pathogen (n = 20). (**b**), net negative effects of increasing *Pseudomonas* genotypic richness on *Pseudomonas* productivity (OD_600_) via intergenotypic bacterial antagonism (n = 19). (**c**), relationship between net effects of *Pseudomonas* intergenotypic antagonism and fungal pathogen inhibition at 2 and 4 genotypic richness treatments (n = 19). *R*^*2*^ calculated from the variance in pathogen inhibition NBE explained by bacterial productivity NBE, independent of species richness effects (Total *R*^*2*^ = 0.48, P < 0.001). All comparisons are made with Analysis of Covariance (see METHODS for details). All points are the mean of three experimental replicates. The values of net biodiversity effects of 8 genotypes are included for visual comparison. See main text for details on how Net Biodiversity Effects (NBE) responses are calculated.

To model the effect of *Pseudomonas* spp. diversity on take-all pathogen growth, we created experimental microcosms and presented the take-all fungal pathogen *in vitro* to 49 different synthetic *Pseudomonas* communities of varying phylogenetic diversity, orthogonally nested within different genotypic richness levels. We characterized the response with the net biodiversity effect^37^: log(Φ_obs_/Φ_exp_), where Φ_obs_ is the overall inhibition by a given assemblage, and Φ_exp_ is the mean inhibition from each genotype present in the assemblage in monoculture. We tested if the ratio of log(Φ_obs_/Φ_exp_) differed between levels of richness and phylogenetic diversity (the null hypothesis being that the ratio remained constant).We found that increasing *Pseudomonas* genotypic richness was associated with a reduced inhibition of the fungal pathogen, beyond that expected from the additive inhibitory effects of individual *Pseudomonas* genotypes (*F*_*1*, *37*_ = 10.45, *P* = 0.003; Fig. 4a). We fitted the presence of each genotype before the genotypic richness term in separate linear models, but none were able to remove the signal of genotypic richness, giving no evidence to suggest a single genotype was completely dominating the communities’ ability to inhibit take-all. We found no influence of *Pseudomonas* phylogenetic diversity on the fungal inhibition net biodiversity effect (*R*^2^ = 0.03; *F*_*1, 37*_ = 1.4, *P* = 0.23; Fig. S3a). We fitted functional diversity in carbon resource use traits in place of phylogenetic diversity, and found it was also a poor predictor of the fungal inhibition net biodiversity effect (*R*^2^ = 0.02; *F*_*1, 37*_ = 0.7, *P* = 0.38; Fig. S4a).

### Bacterial antagonism

We tested the idea that antagonistic interactions between bacterial genotypes increase with richness by conducting a second microcosm experiment. We measured the productivity (OD_600,_) of each genotype when grown on medium that had been cultured by the assemblages we used in the *in vitro* take-all fungal inhibition experiment (Methods). This assay accounts for the compound antagonistic effects of toxin production and nutrient depletion on bacterial growth, which both potentially operate when increasing richness^17^,^20^. Here we define Φ_obs_ as the overall productivity of a genotype on the spent medium from a given assemblage, and Φ_exp_ as the mean productivity of that genotype on the spent medium from each genotype present in that assemblage. As before, we tested if the ratio of log(Φ_obs_/Φ_exp_) differed between levels richness, phylogenetic or functional diversity. We found that *Pseudomonas* community productivity decreased with increasing genotypic richness beyond what would be expected if antagonism between bacterial genotypes did not increase with richness (*F*_*1, 35*_ = 5.6, *P* = 0.024; Fig. 4b). Again, we fitted the presence of each genotype before the genotypic richness term in separate linear models, but as before dominant selection effects by any one genotype were not observed. We were unable to detect any clear signal on the effect of phylogenetic (*R* ^2^= 0.00; *F*_*1, 35*_ = 0.03, *P* = 0.85; Fig. S3b), or functional diversity based on carbon utilization traits (*R*^*2*^ = 0.01; *F*_*1, 35*_ = 0.33, *P* = 0.57, Fig. S4b) on the increases in antagonism between bacteria in mixtures within richness levels. If antagonism between *Pseudomonas* genotypes was an important factor driving fungal inhibition, and if our results were robust across the different media we used on our microcosms, we would have the expectation that the net biodiversity effects observed in our fungal inhibition experiment could be predicted by the variation in the net biodiversity effects in the *Pseudomonas* inhibition experiment. And indeed, we found that the increase in bacterial antagonism in bacterial mixtures was a positively related to the reduction in inhibition of the take-all pathogen by those same bacterial communities (*R* ^2^= 0.29; *F*_*1, 35*_ = 19.56, *P* *< *0.0001; Fig. 4c).

## Discussion

Our field trials show that take-all disease severity, and crop grain losses are positively associated with increased *Pseudomonas* genotypic richness in wheat rhizospheres. Our *in vitro* experiments show that antagonistic interactions between *Pseudomonas* at high genotypic richness can reduce the ability of those bacteria to inhibit growth of the take-all pathogen. This mechanism offers a plausible explanation for our field observations of high take-all disease and crop yield loss in high *Pseudomonas* spp. richness plots. Future work is required to determine the generality of these effects on different media, directly at infection zones on root surfaces, and in different study systems. Our findings support the view that the sign of the biodiversity and ecosystem functioning relationship can be highly context and function specific.

The negative effects of genotypic richness we observed in our model experiments do run contrary to the commonly reported phenomenon that diversity has a positive effect on ecosystem functioning^1^,^40^. Other laboratory experiments have previously shown bacterial antagonism to cause ecosystem functioning to collapse at high richness^17^,^20^, but see Jousset *et al*.^16^. Whilst, (1) the presence of competitively dominant but ‘functionally poor’ species (‘poor’ in a subjective sense, e.g. for a desired function), or (2) the non-additive effects of exploitative and interference competition, are likely to occur in other taxonomic groups, we suggest it may be of limited use to look for general statements of positive vs. negative biodiversity effects. Cautioning against a dichotomy seems important because the direction of the biodiversity ecosystem functioning relationship can depend on both the function and the trophic level of interest. Simply replacing the fungal pathogen in our study with a bacterial pathogen would have likely yielded the opposite results: with bacterial diversity having a positive effect on pathogen control.

Our *in vitro* experiments were designed to orthogonally test for the effects of bacterial phylogenetic (or functional) diversity or genotypic richness on fungal pathogen suppression. We found no effects of phylogenetic functional trait diversity use, but consistent negative effects of genotypic richness, on bacterial antagonism and fungal pathogen suppression. Previous studies have suggested the inability to detect an effect of phylogenetic or functional trait diversity, might result from resource scarcity and limited possibilities for niche partitioning in experimental settings^20^,^22^,^41^. However, it is also possible that (1) core genomes are poor predictors of functional traits due to convergent evolution and horizontal gene transfer^42^; that (2) characterizing functional traits in species grown in isolation are inadequate because trait values can be plastic and change in the presence of different species in mixtures; or (3) simply that trait dissimilarity itself is a poor predictor of niche partitioning^43^.

Our study did not account for local ecological history effects (e.g. specific root location or individual plant clone) which are also likely to influence the biodiversity-ecosystem functioning relationship^19^. Whilst there is evidence to suggest that sympatrically coevolved bacteria can be more antagonistic^44^ there is also evidence to suggest that some background environments enhance positive interactions between species over time^32^. More fine scaled sampling, or real-time imaging of bacterial interactions on the root system, in future could be useful for identifying the impact of isolate sampling location on our results.

It is important to emphasize our field trials were unable to conclude if the negative relationship between *Pseudomonas* spp. genotypic richness and take-all in the field was causal. Sequential sampling of bacterial isolates to map the dynamic interactions between the microbiota of wheat varieties and take-all disease, alongside microbiome transplant experiments and further *in planta* manipulations would help validate this idea. Similarly, although we assayed the most virulent strain of a field isolated take-all pathogen in our model microcosms; we did not assay the effect of different take-all pathogen strains or consider the intraspecific population diversity of the take-all pathogen in our study.

In summary, our findings argue for an integrated view that appreciates the importance of both the negative and positive effects of biodiversity in driving specific ecosystem functions within and across trophic levels. Notably we have focused on the diversity of a single bacterial group, containing putative biocontrol strains highly relevant to our focal fungal pathogen. An analysis of generic groupings of 16S rRNA sequences in the soil at each of our trial plots showed that other bacterial groups are also highly relatively abundant, some of which may play important roles in plant fitness. Testing how co-cultures of larger numbers of isolates from different bacterial genera respond to diversity manipulations, and perform one or more ecosystem functions, remains an important and exciting avenue of research. In particular, future work could identify the applicability of our findings to functions involved in both concurrent disease control and nutrient supply, across multiple bacterial indicator groups and trophic levels. A sharpened focus on these services may help to significantly reduce environmentally and economically costly inputs into global food systems.

## Additional Information

**How to cite this article**: Mehrabi, Z. *et al*. *Pseudomonas* spp. diversity is negatively associated with suppression of the wheat take-all pathogen. *Sci. Rep.* **6**, 29905; doi: 10.1038/srep29905 (2016).

## Supplementary Material

Supplementary Information

## Figures and Tables

**Figure 1:**
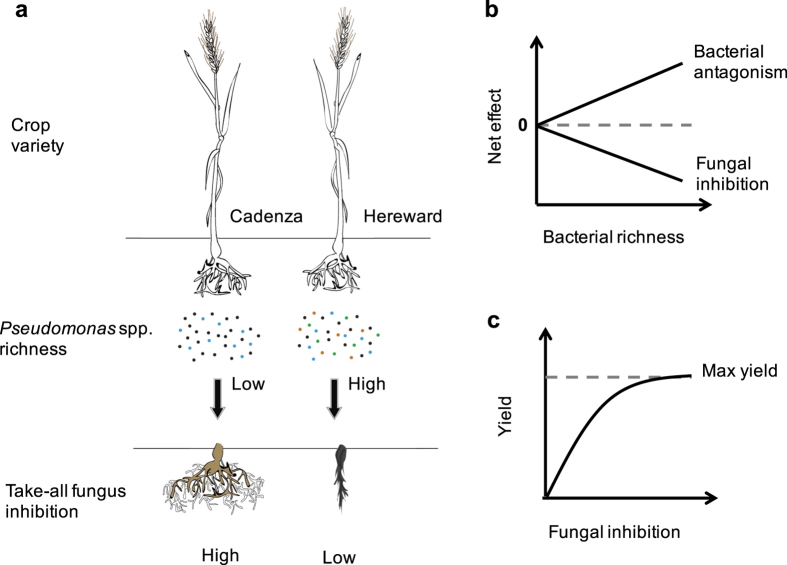
Hypothetical effects of bacterial richness on fungal pathogen inhibition at the plant-soil interface. (**a**), different wheat varieties culture high or low richness of soil bacteria (*Pseudomonas* spp.) which leads to low or high suppression of fungal pathogens in the soil (take-all fungus), respectively. (**b**), as bacterial richness increases, antagonism between bacterial genotypes increases which causes a decrease in ability of the bacterial community to combat fungal pathogens. (**c**), an increase in fungal pathogen inhibition decreases disease incidence and leads to an increase in crop yield.
