# Erratum: *Pseudomonas* spp. diversity is negatively associated with suppression of the wheat take-all pathogen

**DOI:** 10.1038/srep34681

**Published:** 2016-10-10

**Authors:** Zia Mehrabi, Vanessa E. McMillan, Ian M. Clark, Gail Canning, Kim E. Hammond-Kosack, Gail Preston, Penny R. Hirsch, Tim H. Mauchline

Scientific Reports 6: Article number: 29905; 10.1038/srep29905 published online: 10 23 2016; updated: 10 10 2016.

In the Supplementary Information file originally published with this Article, Reference 1 was omitted. This error has now been corrected in the Supplementary Information that now accompanies the Article.

