# Supplementary material for: Pseudomonas spp. diversity is negatively associated with suppression of the wheat take-all pathogen
Source: Sci Rep. 2016 Aug 23;6:29905. doi: 10.1038/srep29905 (PMC4993996; doi:10.1038/srep29905)
Supplement: Supplementary Information [file srep29905-s1.doc]

Title

*Pseudomonas* spp. diversity is negatively associated with suppression of the wheat take-all pathogen

Authors

Zia Mehrabi*[[1]](#footnote-2),2, Vanessa E. McMillan^4^, Ian, M. Clark^3^, Kim E. Hammond-Kosack^4^, Gail Preston^5^, Penny R. Hirsch^3^ & Tim H. Mauchline^3^

Affiliations

^1^Institute for Resources Environment and Sustainability, University of British Columbia, Vancouver, BC Canada V6T 1Z4 ^2^Long Term Ecology Laboratory, University of Oxford, South Parks Road, Oxford, OX1 3PS, UK. ^3^Department of AgroEcology, Rothamsted Research, Harpenden, AL5 2JQ, UK.^4^Department of Plant Biology and Crop Science, Rothamsted Research, Harpenden, AL5 2JQ, UK. ^5^Department of Plant Sciences, University of Oxford, South Parks Road, Oxford, OX1 3RB, UK.

*Author of correspondence

[zia.mehrabi@ubc.ca](mailto:zia.mehrabi@zoo.ox.ac.uk)

+1 604-401-8101

Running header: *Pseudomonas* diversity and take-all build up

Supplementary Information


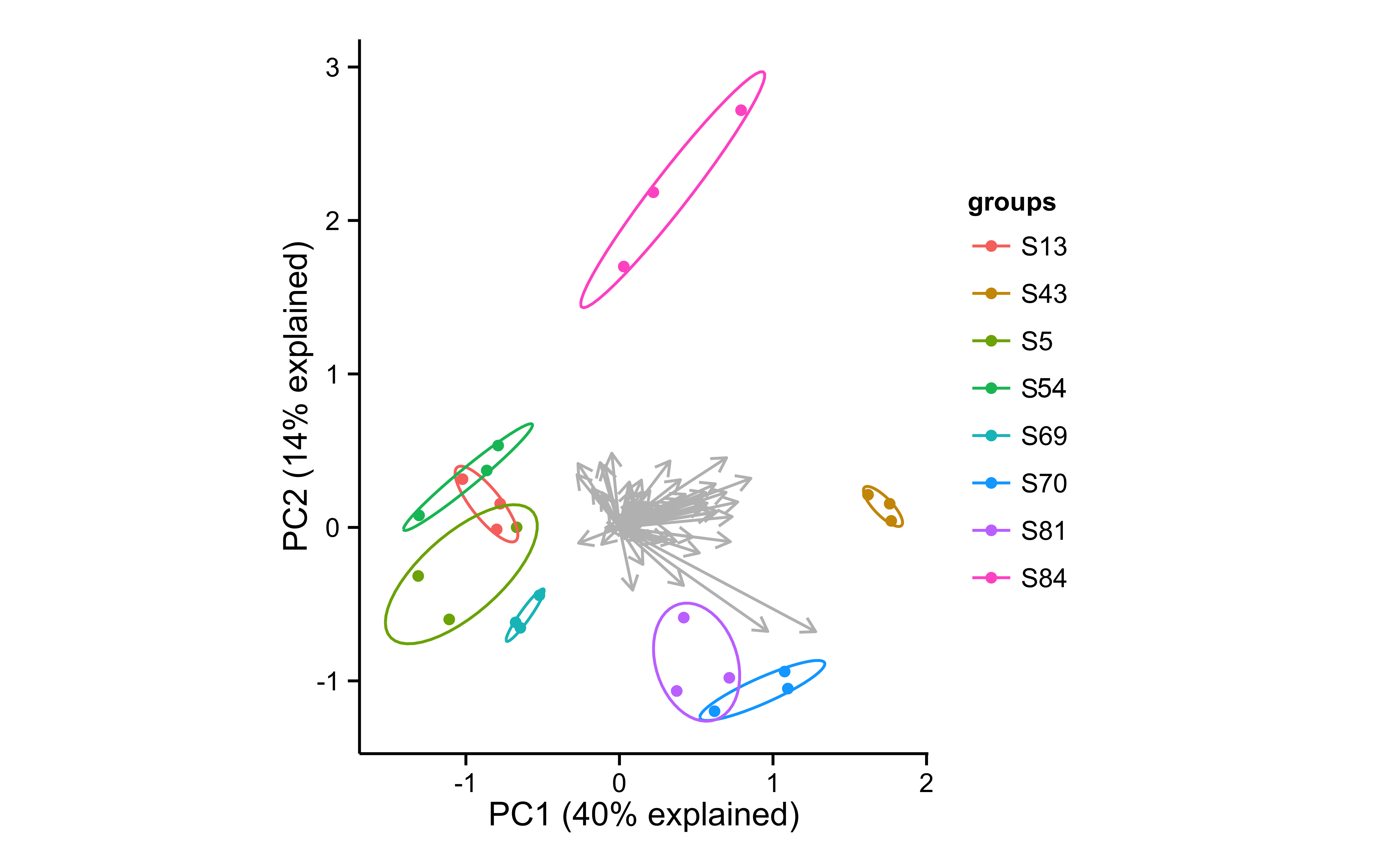


Fig S1 Carbon resource use profile differences between experimental *Pseudomonas* spp. Assays were completed using GN2 MicroPlates (Biolog, Inc.), with 100 µL of each OD_600_ adjusted population per well, incubation at 25°C for 72 h and measurement at OD_590_., in triplicate. Trait values are depicted with a threshold of OD_590_  >0.05. The 8 strains are separated along two major principle components (PC1 and PC2) accounting for 54% of the total variation in carbon use metabolism. Ellipse probability =0.69


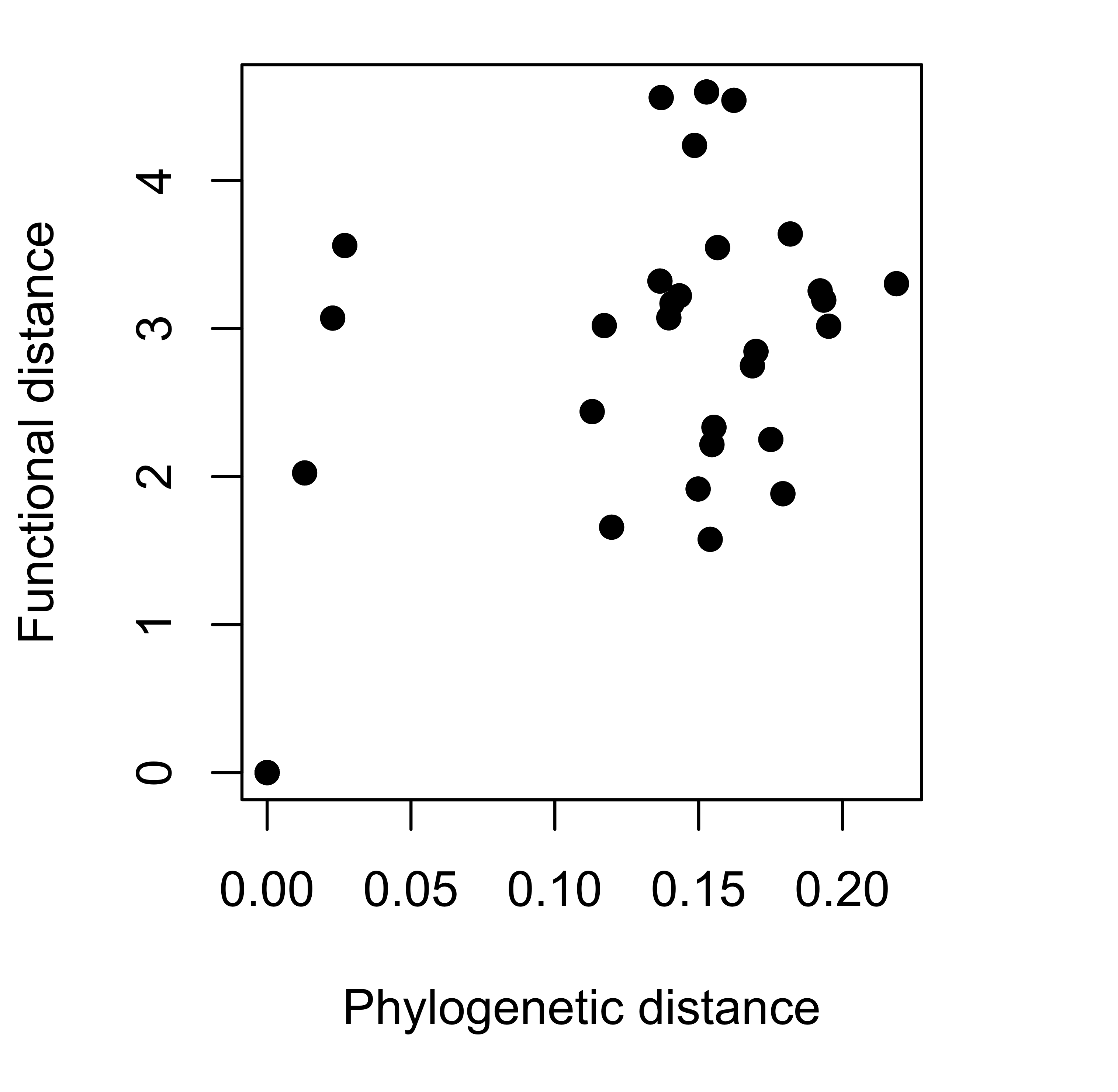


Fig S2. Phylogenetic distance is a poor predictor of the functional distance in carbon use metabolism between experimental *Pseudomonas* genotypes. Phylogenetic distance is based on 940kb *gyrB* fragment phylogeny estimate (Fig 1) and functional distance is based on the euclidean distance between strains for 95 carbon resource traits assayed in Biologg GN2 plates (Fig S1). A Mantel test on the distance matrices was insignificant (*P=*0.74).


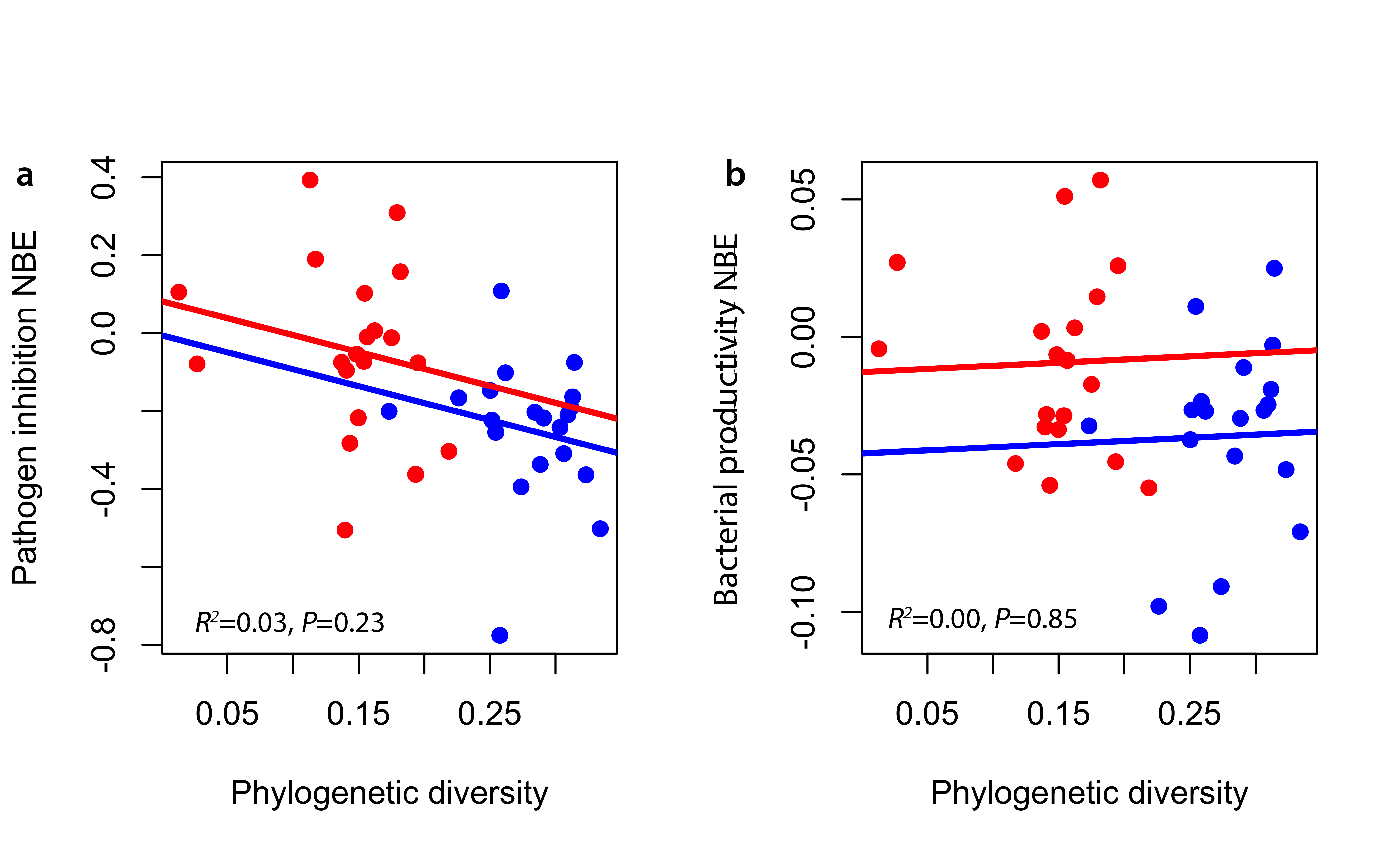


Fig S3 Phylogenetic diversity and net biodiversity effects (NBE) a) Phylogenetic diversity did not influence pathogen inhibition NBE b) Phylogenetic diversity did not influence bacterial productivity NBE. Genotypic richness (catogorical) was fit first and phylogenetic diversity (continuous) second in all models to partition the independent effects of genotypic richness (intercept differences) from phylogenetic diversity (slopes). See main text for calculation of net biodiversity response variables. All analysis was performed in the mean of three experimental replicates (to account for psuedoreplication).*R^2^* values are calculated from the variance explained by phylogenetic diversity, independent of species richness. Red= 2 genotype assemblages; Blue= 4 genotype assemblages.


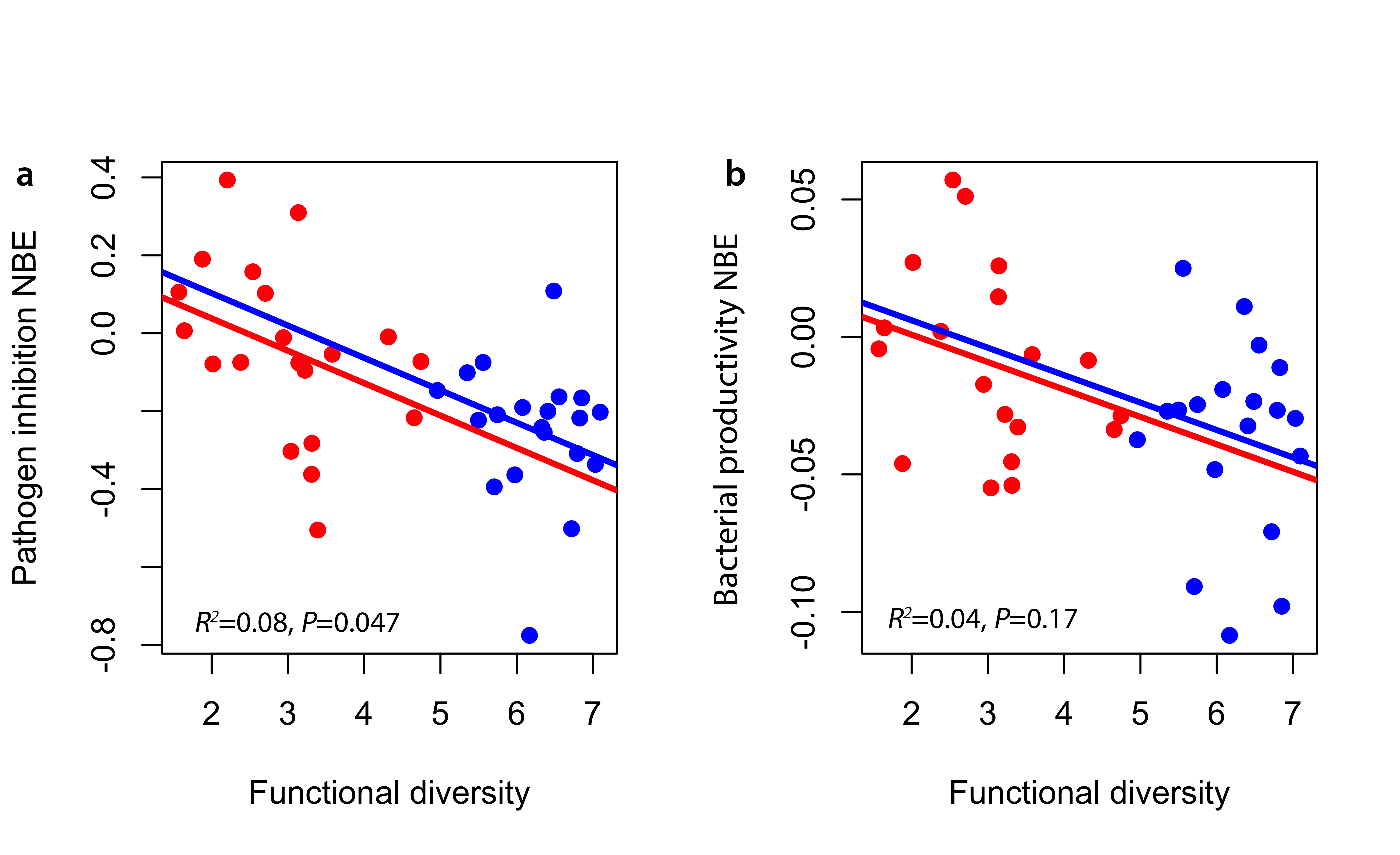


Fig S4 Functional diversity and net biodiversity effects (NBE) a) Functional diversity in carbon resource metabolism (FD) did not influence pathogen inhibition NBE b) FD did not significantly influence negative bacterial productivity NBE. Genotypic richness (catogorical) was fit first and functional diversity (continuous) second in all models to partition the independent effects of genotypic richness (intercept differences) from phylogenetic diversity (slopes). See main text for calculation of net biodiversity response variables. All analysis was performed in the mean of three experimental replicates (to account for psuedoreplication). *R^2^* values are calculated from the variance explained by functional diversity, independent of species richness. All plotted points are the average of three replicates. Red= 2 genotype assemblages; Blue= 4 genotype assemblages


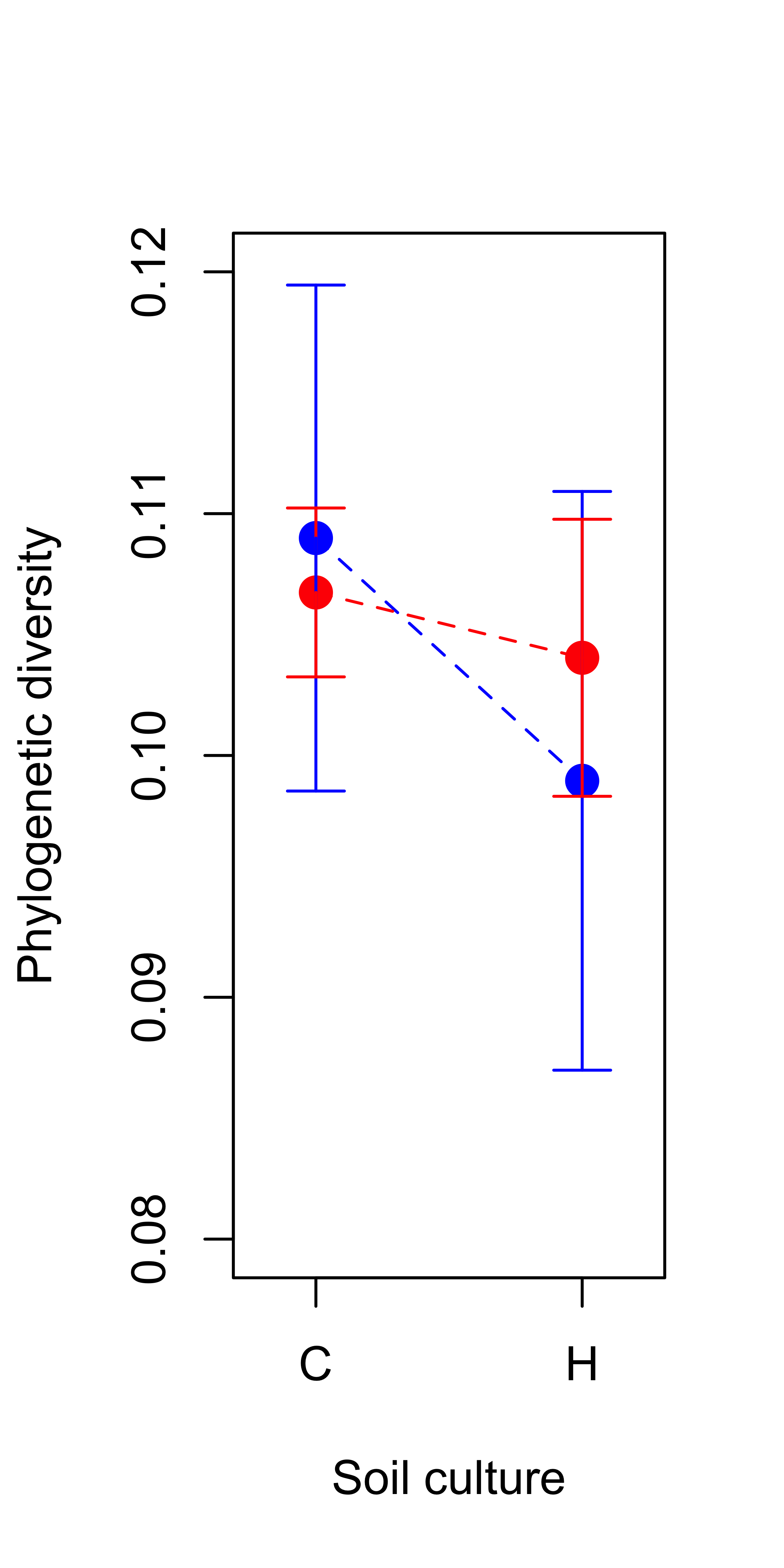


Fig S5 Bacterial phylogenetic diversity and fungal disease load in the field. Mean (±s.e.m) differences in rarefied phylogenetic diversity of *Pseudomonas spp.* between plots with high (H) and low (C) intensity of take-all fungal disease. Blue= endosphere, Red=rhizosphere. All comparisons are non significant (P> 0.05) with Welches t-test. See main text for details. n=4


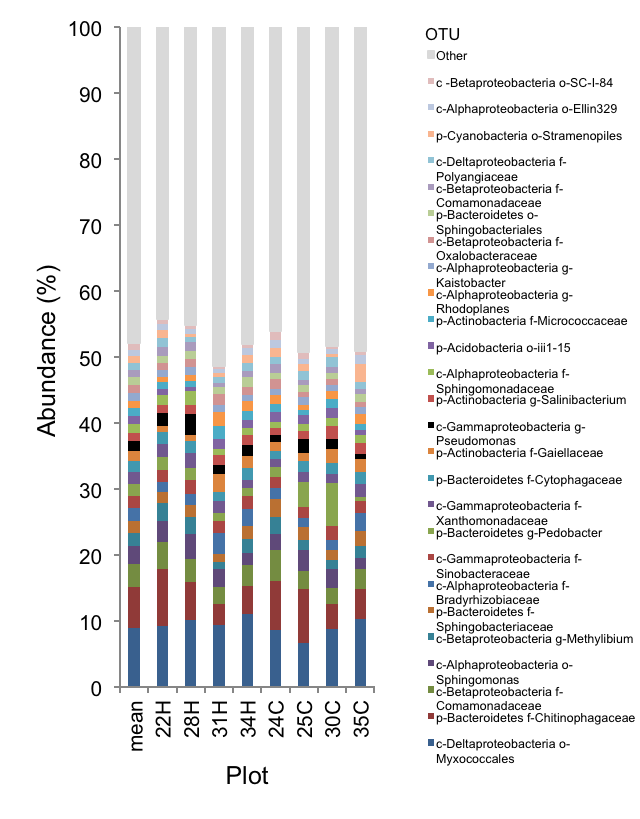


Fig. S6. Comparison of bacterial genera identified by 16S rRNA gene illumina amplicon sequencing in the rhizosphere soil of wheat. This figure shows that *Pseudomonas* spp (in black) are highly prevalent in each of our 8 plots used in the field study. xxH= Hereward, xxC= Cadenza, Mean= mean abundances of all 8 plots. Only operational taxonomic units (OTUs) represented at >1% of total identified amplicons are shown. (see Supplementary Notes 1)

Supplementary Notes 1

These notes describe the methods underlying Fig S6 and highlight the results of microbial community analysis based on 16S rRNA generic groupings. On the 2^nd^ July 2012, 5 x 10cm soil cores were sampled in a W formation in each of the study plots and pooled into 8 samples (one per plot). Bacterial and archaeal 16S rRNA genes were amplified from rhizosphere soil DNA samples with barcoded universal prokaryotic primers (515F/R806) targeting the V4 region, subjected to Illumina® sequencing and analysed using the QIIME 1.8 pipeline (utilizing greengenes for taxa classification and the UCLUST algorithm for OTU clustering), as described by (Caporas*o et a*l., 2010, 2011). After amplicon sequencing 37,788 reads were sub-sampled from each of 8 samples (a total of 302,304 reads). Reads were assigned to bacterial genera (where possible) with 97% sequence identity. We did not observe clear differences in soil community composition between plot treatments based on this amplicon data for the plots in our analyses (ANOSIM: R=0.06 and P=0.37). We found that an average of 1.7% of reads were identified as *Pseudomonas* across the study plots. Lack of sequence variation within the relatively conserved segment of the 16S rRNA gene limit inference to above the level of genus, and so further hypotheses regarding changes in *Pseudomonas* richness, as explored in the main text, cannot be addressed with this amplicon data. However, the results show that *Pseudomonas* comprises an important part of the soil microbiome in all of our plots and that genus based community structure did not differ between the treatments.

Table S1. Community matrix for bacterial isolates sampled from the endosphere and rhizosphere of Xi19 isogenic lines exposed to Hereward and Candenza variety pre-cultures treatments (e.g. see main text “Field Trials” and Fig 2). Unique *Psuedomonas* strains for 8 plots were identified using a 940kb *gyrB* fragment (see Methods). **^§^**Isolates used in the *in vitro* BEF experiments presented in the main text (e.g. Figs 3-4). ^¶^Isolate sampled from the larger *Pseudomonas* library of the Rothamsted Research Great Harpenden 2 Trials (retrieved from the rhizosphere of a Hereward->Hereward plot, not shown here) (see Mauchline et al. 2015). E= Endosphere, R=Rhizosphere.

|  | Hereward -> Xi19 plots | | | | | | | | Cadenza -> Xi19 pls | | | | | | | |
| --- | --- | --- | --- | --- | --- | --- | --- | --- | --- | --- | --- | --- | --- | --- | --- | --- |
| Strain | 22^R^ | 22^E^ | 28^R^ | 28^E^ | 31^R^ | 31^E^ | 34^R^ | 34^E^ | 25^R^ | 2^E^ | 30^R^ | 30^E^ | 35^R^ | 35^E^ | 24^R^ | 24^E^ |
| 3 | 0 | 0 | 1 | 1 | 0 | 0 | 0 | 0 | 0 | 0 | 0 | 0 | 0 | 0 | 0 | 0 |
| 5**^§^** | 0 | 0 | 0 | 0 | 0 | 0 | 0 | 0 | 2 | 0 | 0 | 0 | 0 | 0 | 0 | 0 |
| 7 | 0 | 0 | 1 | 0 | 0 | 0 | 1 | 0 | 0 | 0 | 0 | 0 | 0 | 0 | 0 | 0 |
| 9 | 0 | 0 | 1 | 0 | 0 | 0 | 0 | 0 | 0 | 0 | 0 | 0 | 0 | 0 | 0 | 0 |
| 10 | 0 | 0 | 1 | 0 | 0 | 0 | 0 | 0 | 0 | 0 | 0 | 0 | 0 | 0 | 0 | 0 |
| 11 | 0 | 0 | 1 | 0 | 0 | 0 | 0 | 0 | 0 | 0 | 0 | 0 | 0 | 0 | 0 | 0 |
| 12 | 0 | 0 | 1 | 0 | 0 | 0 | 0 | 0 | 0 | 0 | 0 | 0 | 0 | 0 | 0 | 0 |
| 13**^§^** | 0 | 0 | 0 | 2 | 0 | 0 | 0 | 0 | 0 | 0 | 2 | 0 | 0 | 0 | 0 | 0 |
| 14 | 0 | 0 | 1 | 7 | 0 | 0 | 0 | 0 | 0 | 0 | 0 | 0 | 0 | 0 | 0 | 0 |
| 15 | 0 | 0 | 1 | 1 | 0 | 0 | 0 | 0 | 0 | 0 | 0 | 0 | 0 | 0 | 0 | 0 |
| 16 | 0 | 0 | 1 | 0 | 0 | 0 | 0 | 0 | 0 | 0 | 1 | 0 | 0 | 0 | 0 | 0 |
| 17 | 0 | 0 | 0 | 0 | 0 | 0 | 0 | 0 | 0 | 0 | 0 | 2 | 0 | 0 | 0 | 0 |
| 18 | 0 | 0 | 0 | 0 | 0 | 0 | 0 | 0 | 0 | 0 | 2 | 8 | 0 | 0 | 0 | 0 |
| 21 | 4 | 4 | 0 | 0 | 0 | 0 | 0 | 0 | 0 | 0 | 0 | 0 | 0 | 0 | 0 | 0 |
| 33 | 1 | 0 | 0 | 0 | 0 | 0 | 1 | 0 | 0 | 0 | 0 | 0 | 0 | 0 | 0 | 0 |
| 34 | 3 | 1 | 0 | 0 | 0 | 0 | 0 | 0 | 0 | 0 | 0 | 0 | 0 | 0 | 0 | 0 |
| 35 | 3 | 2 | 0 | 0 | 0 | 0 | 0 | 0 | 0 | 0 | 0 | 0 | 0 | 0 | 0 | 0 |
| 36 | 1 | 2 | 0 | 0 | 0 | 0 | 0 | 0 | 0 | 0 | 0 | 0 | 0 | 0 | 0 | 0 |
| 37 | 1 | 1 | 0 | 0 | 0 | 0 | 0 | 0 | 0 | 0 | 0 | 0 | 0 | 0 | 0 | 0 |
| 40 | 0 | 0 | 0 | 0 | 1 | 0 | 0 | 0 | 0 | 0 | 0 | 0 | 0 | 0 | 0 | 0 |
| 41 | 0 | 0 | 0 | 0 | 2 | 0 | 0 | 0 | 0 | 0 | 0 | 0 | 0 | 0 | 0 | 0 |
| 44 | 0 | 0 | 0 | 0 | 1 | 0 | 0 | 0 | 0 | 0 | 0 | 0 | 0 | 0 | 0 | 0 |
| 45 | 0 | 0 | 0 | 0 | 1 | 0 | 0 | 0 | 0 | 0 | 0 | 0 | 0 | 0 | 0 | 0 |
| 46 | 0 | 0 | 0 | 0 | 1 | 0 | 0 | 0 | 0 | 0 | 0 | 0 | 0 | 0 | 0 | 0 |
| 47 | 0 | 0 | 0 | 0 | 2 | 0 | 0 | 7 | 0 | 0 | 0 | 0 | 0 | 0 | 0 | 0 |
| 48 | 0 | 0 | 0 | 0 | 0 | 0 | 7 | 0 | 0 | 0 | 0 | 0 | 0 | 0 | 0 | 0 |
| 54**^§^** | 0 | 0 | 0 | 0 | 0 | 0 | 0 | 5 | 0 | 0 | 0 | 0 | 0 | 0 | 3 | 0 |
| 69**^§¶^** | 0 | 0 | 0 | 0 | 0 | 0 | 0 | 0 | 0 | 0 | 0 | 0 | 0 | 0 | 0 | 0 |
| 70**^§^** | 0 | 0 | 1 | 0 | 3 | 1 | 2 | 0 | 1 | 0 | 1 | 0 | 0 | 0 | 6 | 0 |
| 71 | 0 | 0 | 0 | 0 | 0 | 0 | 0 | 0 | 0 | 0 | 0 | 0 | 0 | 0 | 2 | 0 |
| 74 | 0 | 0 | 1 | 0 | 1 | 0 | 2 | 0 | 0 | 0 | 0 | 0 | 0 | 0 | 0 | 0 |
| 81**^§^** | 0 | 0 | 0 | 0 | 0 | 0 | 0 | 0 | 0 | 0 | 0 | 0 | 0 | 0 | 0 | 2 |
| 82 | 0 | 0 | 0 | 0 | 0 | 0 | 0 | 0 | 0 | 0 | 0 | 0 | 0 | 0 | 0 | 1 |
| 84**^§^** | 0 | 0 | 0 | 0 | 0 | 0 | 0 | 0 | 0 | 0 | 0 | 0 | 0 | 0 | 0 | 2 |
| 86 | 0 | 0 | 0 | 0 | 0 | 0 | 0 | 0 | 0 | 0 | 0 | 0 | 0 | 0 | 0 | 1 |
| 87 | 0 | 0 | 0 | 0 | 0 | 0 | 0 | 0 | 0 | 0 | 0 | 0 | 0 | 0 | 0 | 1 |
| 89 | 0 | 0 | 0 | 0 | 0 | 0 | 0 | 0 | 0 | 0 | 0 | 1 | 0 | 0 | 0 | 0 |
| 50 | 0 | 0 | 0 | 0 | 0 | 0 | 0 | 0 | 0 | 0 | 0 | 0 | 2 | 0 | 0 | 0 |
| 83 | 0 | 0 | 0 | 0 | 0 | 0 | 0 | 0 | 0 | 0 | 0 | 0 | 0 | 0 | 0 | 4 |
| 43**^§^** | 0 | 0 | 0 | 0 | 1 | 6 | 1 | 0 | 9 | 12 | 3 | 0 | 1 | 0 | 0 | 0 |
| 85 | 0 | 0 | 0 | 0 | 0 | 0 | 0 | 0 | 0 | 0 | 0 | 0 | 0 | 0 | 0 | 2 |
| 52 | 0 | 0 | 0 | 0 | 0 | 0 | 0 | 0 | 0 | 0 | 0 | 0 | 0 | 1 | 0 | 0 |
| 51 | 0 | 0 | 0 | 0 | 0 | 0 | 0 | 0 | 0 | 0 | 0 | 0 | 6 | 9 | 0 | 0 |

1. [↑](#footnote-ref-2)
